# Editorial: Advances in PET-CT imaging, vol. II

**DOI:** 10.3389/fmed.2026.1957995

**Published:** 2026-08-17

**Authors:** Giulia Santo, Maria Gazzilli, Francesco Dondi

**Affiliations:** 1 Department of Nuclear Medicine, Medical University of Innsbruck, Innsbruck, Austria; 2 Department of Nuclear Medicine, ASL Bari-P.O. Di Venere, Bari, Italy; 3 Department of Nuclear Medicine, ASST Spedali Civili di Brescia, University of Brescia, Brescia, Italy

**Keywords:** 18F-FDG, disease detection, PET-CT, quantification, radiopharmaceuticals

**Figure 1:**
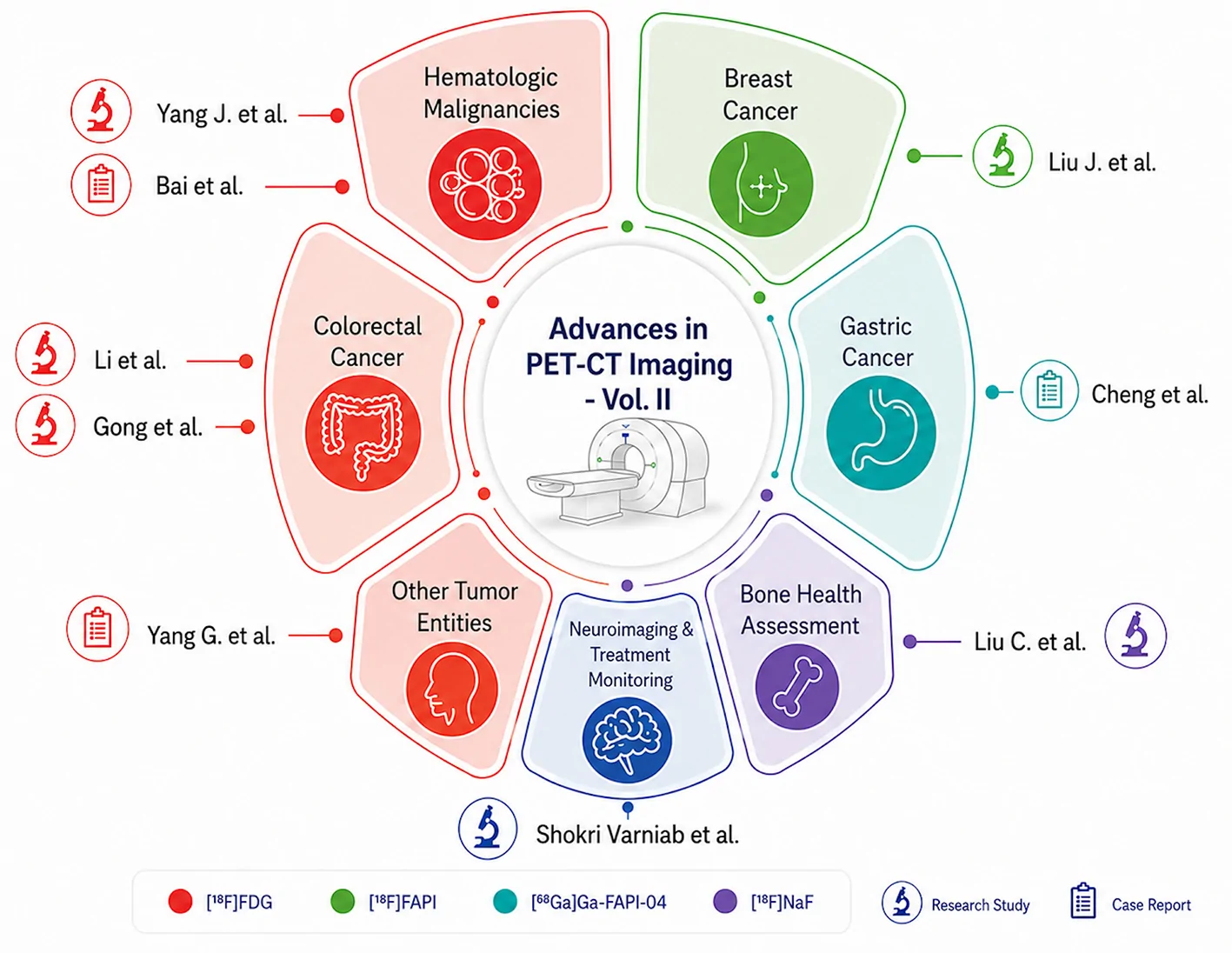
Schematic overview of the studies included in the Research Topic “*Advances in PET-CT imaging, vol. II*,” illustrating the broad spectrum of clinical applications, radiopharmaceuticals, and disease settings covered by this Research Topic.

Following the success of the first volume of the Research Topic “*Advances in PET-CT Imaging*” (1), we are pleased to introduce this second edition, which brings together nine contributions, including six original research articles and three case reports. These studies provide an overview of the current landscape of PET/CT imaging, highlighting not only its well-established role in oncology but also its expanding applications across a broad spectrum of clinical settings. Figure 1 provides a graphical summary of the content of this Research Topic.

Although the introduction of novel tracers has considerably expanded the horizons of molecular imaging, [^18^F]FDG PET/CT remains the cornerstone of oncological PET imaging. Several contributions included in this Research Topic further refine our understanding of its diagnostic potential. In the field of hematological malignancies, Yang J. et al. investigated the characteristics of newly detected lesions on interim or end-of-treatment [^18^F]FDG PET/CT in 20 patients with different lymphoma subtypes. Their study demonstrated that quantitative PET parameters, particularly the maximum standardized uptake value (SUV_max_) and the tumor-to-background ratio (TBR), represent valuable imaging biomarkers for differentiating lymphoma-related lesions from benign findings. These observations may improve image interpretation and reduce the number of false-positive assessments, supporting more accurate therapeutic decision-making. In this scenario, Bai et al. described a patient affected by angioimmunoblastic T-cell lymphoma (AITL). In this case report, the authors highlight how this rare lymphoma subtype may exhibit false-negative [^18^F]FDG PET/CT findings, potentially delaying the diagnostic process. Their experience emphasizes that, when clinical suspicion remains high despite inconclusive PET findings, a comprehensive multimodal diagnostic approach including clinical evaluation, laboratory biomarkers, flow cytometry, and careful biopsy site selection remains essential to establish the correct diagnosis.

The usefulness of [^18^F]FDG PET/CT also extends to colorectal cancer. In a retrospective study including patients with colorectal carcinoma and colorectal adenoma, Li et al. showed that volumetric metabolic parameters, particularly metabolic tumor volume (MTV) and total lesion glycolysis (TLG), outperform conventional SUV-based metrics in distinguishing malignant from benign colorectal lesions. These findings reinforce the growing importance of volumetric PET metrics, which may contribute to more accurate lesion characterization. The diagnostic value of [^18^F]FDG PET/CT in colorectal cancer was

further explored by Gong et al., who compared PET/CT with whole-body bone scintigraphy using SPECT for the detection of bone metastases. Their results clearly demonstrated the superior diagnostic performance of PET/CT, which achieved substantially higher sensitivity, specificity, and overall diagnostic accuracy. The relevance of [^18^F]FDG PET/CT is also reflected in the diagnosis of other tumor entities. Namely, the case reported by Yang G. et al. highlights the pivotal contribution of PET/CT in the evaluation of oral mucosal melanoma. By accurately defining the extent of disease, PET/CT demonstrated its value as an essential component of multidisciplinary oncological management, even in rare clinical scenarios.

While [^18^F]FDG remains the reference standard for most oncological indications, the continuous development of novel radiopharmaceuticals is rapidly reshaping the landscape of molecular imaging. Among these, fibroblast activation protein inhibitor (FAPI)-based tracers have emerged as one of the most promising tracers of recent years. Two contributions included in this Research Topic further illustrate the expanding role of this imaging approach. In a retrospective single-center study, Liu J. et al. evaluated the diagnostic performance of [^18^F]AlF-NOTA-FAPI-04 PET/CT in 69 therapy-naïve patients with suspected breast cancer, comparing its performance with that of conventional multimodality imaging, including ultrasonography, mammography, breast magnetic resonance imaging, computed tomography, and bone scintigraphy. Although conventional imaging maintained excellent accuracy for the assessment of primary breast lesions, FAPI PET/CT demonstrated clear advantages in the evaluation of regional lymph node involvement. These findings suggest that FAPI PET/CT may complement rather than replace conventional breast imaging, providing additional information in those clinical scenarios where accurate staging is most likely to influence therapeutic decision-making. The complementary value of FAPI imaging is further highlighted by the case report presented by Cheng et al. A patient with progressive gastric outlet obstruction, marked weight loss, and persistent clinical suspicion of gastric cancer repeatedly underwent endoscopic biopsies that revealed only chronic inflammatory changes. Whereas conventional [^18^F]FDG PET/CT demonstrated only mild uptake, [^68^Ga]Ga-FAPI-04 PET/CT identified intense tracer accumulation corresponding to the infiltrative gastric lesion, guiding definitive surgical management. Histopathological examination confirmed a poorly differentiated gastric adenocarcinoma with signet-ring cell features.

However, the potential of PET/CT is not limited to cancer characterization. An example of this evolving perspective is provided by Liu C. et al., who explored the feasibility of [^18^F]NaF PET/CT as a comprehensive tool for opportunistic bone health assessment in patients with prostate cancer without skeletal metastases. By combining low-dose CT-derived trabecular attenuation with quantitative measurements of fluoride uptake, the authors demonstrated a strong correlation between structural bone density and bone metabolism while identifying metabolically discordant phenotypes that would not be evident using anatomical imaging alone. The study also suggests that the added clinical value of [^18^F]NaF PET/CT may be greatest in patients with indeterminate structural findings, where metabolic information could refine risk stratification beyond conventional CT assessment.

The expanding role of PET imaging is equally evident in the field of neuroimaging. In a longitudinal PET/MRI study, Shokri Varniab et al. investigated cerebral metabolic changes following high-dose methotrexate treatment in pediatric and young adult patients with cancer. Their findings revealed a dynamic pattern of brain metabolism characterized by an early increase in cortical [^18^F]FDG uptake shortly after treatment, followed by normalization or mild hypometabolism during later follow-up. Although none of the patients developed clinically evident neurological toxicity, these metabolic alterations likely reflect evolving biological processes occurring before the appearance of structural abnormalities or neurological symptoms. Thus, molecular imaging may provide sensitive biomarkers for monitoring treatment effects long before irreversible damage becomes clinically manifest.

To sum up, the studies collected in this Research Topic highlight the significant advances in PET/CT imaging. The growing understanding of [^18^F]FDG PET/CT, together with the progressive introduction of novel radiopharmaceuticals and advances in quantitative imaging and disease detection, represents a promising perspective for consolidating PET/CT as an essential tool across a wide range of clinical scenarios.
